# “I just need to be with my family”: resettlement experiences of asylum seeker and refugee survivors of torture

**DOI:** 10.1186/s12992-021-00681-9

**Published:** 2021-03-09

**Authors:** Kim S. Griswold, Bonnie M. Vest, Angelique Lynch-Jiles, Douglas Sawch, Kateryna Kolesnikova, Leonce Byimana, Pamela Kefi

**Affiliations:** 1 Jacobs School of Medicine and Biomedical Sciences, SUNY at Buffalo, Primary Care Research Institute, Buffalo, USA; 2 School of Public Health and Health Professions, State University of New York at Buffalo, Buffalo, USA; 3 The Jacobs School of Medicine and Biomedical Sciences, State University of New York at Buffalo, Buffalo, USA; 4 Center for Survivors of Torture, Jewish Family Service of Buffalo and Erie County, Buffalo, New York USA; 5 Buffalo, NY USA

**Keywords:** Refugees, Asylum seekers, Resettlement, Torture, Mental health, Trauma informed care

## Abstract

**Abstract:**

A global migration of individuals fleeing persecution, violence and armed conflict reached almost 60 million world-wide in 2015. This world-wide crisis of displacement reflects people seeking safety across borders and oceans; dangerous journeys that compound the trauma endured by these women, men and children. Refugees/asylum seekers face barriers upon entry to the U.S. The Western New York Center for Survivors (WNYCST) provides care coordination/trauma-informed care to mitigate these challenges. The objective of this study was to explore the resettlement experiences of survivors of torture living in Western New York, who had received services from the WNYCST; identifying challenges, unmet needs, and services that were helpful. Secondarily, we describe the experiences of asylum seekers and legally resettled refugees, who due to their differing legal status, might be expected to have different experiences. Data were collected using semi-structured qualitative interviews.

**Results:**

Three themes emerged: mental health challenges, relating to their experiences in their home country and their separation from family; unmet needs, including lack of a sense of purpose and meaning, difficulty navigating services, and missing connections to community; and coping strategies, including WNYCST assistance with connecting with sources of social support in their new community. WNYCST services were helpful, particularly the assistance and connection with care coordinators and local support groups. This care and outreach helped to mitigate feelings of separation and apartness from their home countries and families.

**Conclusions:**

Some refugees/asylum seekers continue to struggle with unmet needs, issues of loss and isolation. If care providers recognize signs of stress early, appropriate interventions can be implemented. Care connections and trauma informed treatment with an emphasis on recreating ties with communities, may be one important factor in ensuring successful integration.

## Background

A global migration of individuals fleeing persecution, violence and armed conflict reached almost 60 million world-wide in 2015 [1]. This world-wide crisis of displacement reflects people seeking safety across borders and oceans; dangerous journeys that compound the trauma endured by these women, men and children [2]. A hoped for outcome by many is the attainment of asylum in different countries around the world. As defined by the Human Rights Convention and Refugee Act of 1951, refugees are people who are unable or unwilling to return to their country of origin based on a well-founded fear of persecution due to race, religion, nationality, political opinion or membership in a particular social group [3]. Refugees are screened for resettlement *outside* the U.S., whereas asylum seekers make an application for asylum once actually *present* in the U.S. There are differences for both groups in admission processes, and in the agencies responsible for their application review [4]. Both refugees and asylum seekers face adjustment and resettlement challenges upon entry to the U.S. Paramount are feelings of loss and displacement, fear of the new situations they face, and for asylum seekers, uncertainty about their future. These issues are exacerbated among survivors of torture and persecution [5, 6].

Estimated prevalence rates of torture among refugees and asylum seekers vary greatly, as much as from 2.7 to 100%, based upon the study and the sample characteristics [7]. A systematic review conducted in 2009 found a prevalence rate of torture across 84 studies of 21% [8]. Further, their meta-analysis demonstrated that torture was a significant contributor to PTSD and depression, after adjusting for methodological variance across studies [8]. Similarly, the experience of a greater number of torture exposures and types is also predictive of higher risk of both anxiety and PTSD [9].

Western New York, and Erie County in particular, resettles approximately 4500 refugees annually, with an additional number of individuals seeking asylum [10]. Given the high level of need and risk for adverse outcomes among survivors of torture, the Western New York Center for Survivors of Torture (WNYCST) was developed in 2014 to address the trauma experienced by refugees and asylum seekers by providing care coordination, trauma-informed treatment, and holistic management for individuals and families. Survivors are identified by local resettlement agencies, legal service providers, and community organizations, and are referred to the WNYCST for assessment and care coordination. Care coordination activities are client-centered and address the full spectrum of needs, including: physical and mental health care, social services, housing, child care, transportation, language and communication, employment, community engagement, and legal needs. The WNYCST served 57 clients from October 1, 2015 – September 30, 2016, during the time of this study.

The primary objective of this study was to explore the resettlement experiences of survivors of torture living in Western New York, who had received services from the WNYCST; identifying challenges, unmet needs, and services that were helpful. Secondarily, based upon the findings of the qualitative thematic analysis, we also describe some of the identified differences in the experiences of asylum seekers and legally resettled refugees, who due to their differing legal status, might be expected to have different experiences.

## Methods

### Recruitment

To be considered eligible, participants had to be clients of the WNYCST who self-identified as survivors of torture, and be willing and able to consent to participation in the interview. WNYCST staff identified a convenience sample of clients who might be eligible for an interview, provided them with information on the interview, and made arrangements (date, time, location, and interpretation as needed) for the client to meet with the research staff. All clients approached by the WNYCST staff agreed to participate.

### Study team

The study team who collected and analyzed the data consisted of: a family medicine physician (KG), who also served as the medical director for the WNYCST and conducts forensic examinations to document torture and trauma among asylum seekers; a medical anthropologist (BV) with expertise in qualitative research methods who led program evaluation for the WNYCST; and three medical students (DS, KK, ALJ) who were volunteering with the WNYCST as part of the University at Buffalo’s Human Rights Initiative.

### Participants

A total of 15 clients of the WNYCST consented to participate in the interviews. Eight (53.3%) participants were female. Nine (60%) of the participants were married. The average age of participants was 42.6 (SD: 8.3, range 29–61). Clients came from 7 different countries, including: Angola, Democratic Republic of the Congo, Eritrea, Iraq, Myanmar, Nigeria, and Rwanda, but the largest group of interviewees (47.7%; *n* = 7) were from Iraq and identified their primary language as Arabic. Other primary languages included: French, Lingala, Burmese and Kinyarwanda. Length of time in the United States at the time of the interview ranged from six months to seven years, with most participants (*n* = 10) identifying length of approximately 1–4 years. Six participants were asylum seekers, six were resettled refugees, one had a special immigrant visa, and two had unknown immigration status. Five participants were employed at the time of the interview, working primarily in service industries such as housekeeping, delivery, and tailoring; the remainder were unemployed. Participants’ formal education ranged from none to college-level (6 middle school or less, 2 high school, 5 college-level education, and 2 unknown).

## Materials and procedure

### Data collection

Data were collected using semi-structured qualitative interviews. Interview questions focused on clients’ resettlement experiences, interactions with WNYCST staff and services, and continued unmet needs. The first and second author created the interview guide, and questions were shared with WNYCST leadership and care coordinators for review and vetting, prior to being used with clients. The interviews contained no questions related to clients’ trauma or torture history. In the event of a client seeming uncomfortable or upset, case managers and mental health workers were available on site. Interviews were conducted by a medical anthropologist and two medical students in two waves, in the summers of 2015 and 2016. All medical students received training in the interview process, including identification of client stress; all had experience in working with interpreters. For participants who did not speak English, trained interpreters from either the WNYCST, or a telephonic service were used. Participants who were conversant in English were interviewed in English without an interpreter. Interviews were audio recorded for all participants, except one who declined to be recorded. Handwritten notes were taken for that interview. All interviews were transcribed by the medical students who conducted the interviews.

All participants provided verbal informed consent to participate in the project. Research staff read a study information sheet containing all of the information required in a consent form. Participants were told their participation was voluntary and would not affect services they received at the WNYCST and that their comments would not be reported back to their case managers. Participants consented both to participate in the interview and to be recorded. The study protocol was approved by the Institutional Review Board at the University at Buffalo.

### Data analysis

Data were analyzed using a thematic content analysis approach [11, 12]. Two researchers independently reviewed the transcripts and identified themes, or ideas that were expressed repeatedly across transcripts. They then met to confer and compare themes and categorization and resolve any discrepancies. All identified themes were combined into a master list, and then reviewed again to ensure agreement with the final organization. Once this was finalized, the transcripts were reviewed to identify exemplary quotations for each theme.

## Results

Participants’ discussions of their experiences fell into three main themes: 1) mental health challenges, relating to their experiences in their home country and their separation from family; 2) unmet needs, including lack of a sense of purpose and meaning, difficulty navigating services, and missing connections to community; and 3) social support, including WNYCST assistance with connecting with resources in their new community.

### Mental health challenges

Clients mentioned many challenges related to resettling in the United States, including mental and physical health challenges that made it difficult for them to work, leave their home, or learn English. Many participants discussed having problems with their memory. Participants mentioned being unable to remember past dates and events, with the exception of the torture they experienced. One participant described, through the translator:

*“The only wish for her [the participant] that she forget the nine days when she was kidnapped. And she said she have a really bad memory and she forget everything but these nine days are the only things that she cannot forget.”* [Participant #13, Female, Iraq].

Memory problems also made it difficult for clients to settle into life in the United States; participants expressed challenges with learning English and other new things, because they would forget everything they learned.

This also related to mental health challenges. Participants discussed always feeling their mind was busy, or unsettled, and being unable to focus, except on bad past memories or worry about their family members overseas. For example, one asylum seeker from Nigeria said “*This is my life since 2008, I have this problem you know, I never have a relaxed mind, I’m living in fears.*” [Participant #2, Female, Nigeria].

Separation from family was especially difficult, and likely exacerbated and contributed to mental health difficulties faced by survivors. Participants expressed struggles with the psychological and emotional difficulties of being separated from family and children who were still back in their home country. Emotions ranged from guilt over leaving family behind, to fear and worry about their safety and coping was difficult due to uncertainty about whether family members would be able to join them in the U.S.

“*What give her hope that she, even if she’s suffering, but she believes that when her kids will be able to come here, they will have a better life...they don’t know how they gonna do this [bring kids]. Very complex*.” [Participant #12, Female, Angola].

Asylum seekers in particular expressed mental health difficulties relating to the uncertainty of their legal status in the U.S. They discussed a feeling that their life was on hold, or that they could not really settle into their new life until they knew the outcome of their asylum case.

*“[My care coordinator gave me] advice for my life because I did have much trouble in my mind…because my case is pending and I don’t have green card…some day I was like losing my mind.”* [Participant #11, Male, Angola]*.*

They also discussed feeling worried because they were unable to help their families until they had a decision on their own case, and a frustration over the long timeframes involved in obtaining a decision. Participant #11 discussed how his wife had come from Angola to be with him, because she was having problems, but their children are still in Angola and he is more worried about them now, knowing a parent is not with them. However, he was uncertain if he could bring them to the U.S. as well,

*“I don’t see the way I can make that because everything depend off my case. My case is pending. Nobody can move.”* [Participant #11, Male, Angola].

Another participant discussed,

*“It takes so many years to get your work permit. This is the main problem. I know people who wait for 6 years to get permit. Sometimes need to make trip as soon as possible but have to wait for several years to be reunited with your family. Sometimes people just say, let me go back home, I don’t care about case, I just need to be with my family.”* [Participant 5, Male DRC].

### Unmet needs

In terms of unmet needs, clients discussed wanting to be productive and obtain employment, especially in the field in which they worked in their home countries. Asylum seekers in particular expressed difficulty with feeling that they didn’t have a purpose or meaning to their life in the United States. Being idle was difficult and some participants expressed a sense that having work would make them feel better mentally as well. As one male participant from the DRC said,

*“Most important thing is to start as soon as possible to start with work, so can be useful for society…must wait for several months before can go for papers/case. While waiting, you are inactive, just waiting…Let’s do something so that they can be active while they are waiting.”* [Participant #5, Male, DRC].

Another said, *“In my life, I like to work…every day I need to work. When I said I’m stay here, I’m starting to find out application for asylum, they told me after 6 months- after 5 months, I can apply for work permit. After I apply work permit, I am waiting….then, 180 days, 6 month again, you see? …I have a really really hungry for the work, for the job.”* [Participant #8, Female, Rwanda].

Participants also expressed difficulties with navigating the system in the United States, untangling programs and services and uncertainty over what help they could ask from different programs and individuals. For example, one Iraqi woman discussed that she needed help with applying for food stamps, but had not asked her care coordinator for help because, “*She had more stuff that she always talk about…and she feel that, she scared that it’s going to be too much to ask for.*” [Participant #10, Female, Iraq]. One participant described how she felt disempowered in the U.S., where she knew how to advocate and get help in her home country,

*“She has a lot of problem. Like she wouldn’t know even where to go here…she said back home she would know where to go and scream and yell and do something…but here she just don’t know what to do and where to go, and even if she wanted to go to a government or somebody in charge, she wouldn’t know where and how to do that.”* [Participant #9, Female, Iraq].

Finally, participants expressed a sense of isolation in the U.S. and a desire for more community and social engagement. However, some expressed concern about interacting with others from their home country. This was particularly evident among the Iraqi refugees, some of whom did not want to be with other Iraqis or have them learn their story. This may have been partly due to religious differences among Iraqi refugees. Because of this, and other mental and emotional challenges, participants discussed feeling isolated, having difficulty leaving their home, keeping appointments, and meeting others. Many were dependent on others for transportation which limited their mobility.

*“I only know [the case managers]…I don’t know nobody in nowhere, I just know the office [WNYCST].”* [Participant #4, Female, Eritrea].

### Coping strategies

In spite of all these difficulties, clients expressed gratitude for the WNYCST. Care coordinators helped survivors overcome some of the challenges related to mental health and separation from family and their home country by connecting them to the community. Often, participants spoke of how they had come to view the care coordinators as family and rely on them for emotional, spiritual and instrumental support,

*“She said that when she comes here [to the WNYCST], she feels like she is coming to visit her family and she feels like home when she is here.*” [Participant #10, Female, Iraq].

Participants indicated care coordinators were helpful with meeting neighbors, finding churches, and locating support group programs. One Iraqi woman was particularly grateful to WNYCST for connecting her with some white American women who have become her friends, take her to church, and help her to learn English.

*“She says her relationship with American people kind of give her that comfort from the stress that she get from the Iraqi community...she says [her friend] pick them up every time, every week, and take them to the church and bring them back, she said. But she’s doing that because she really loves them.”* [Participant #13, Female, Iraq].

Participants also found comfort in peer support groups, and one woman’s group in particular, for victims of human trafficking, was mentioned by a few clients as being helpful. This group offered faith-based activities, cooking classes, dancing, and taught participants about self-care. Participants also expressed receiving assistance from other communities of refugees living locally in the area, who help them find and furnish homes, and assist with transportation and language.

Finally, participants discussed how maintaining a positive attitude and having goals for their lives, to obtain employment, buy a house, and reunite with their families helped them to feel better.

“*I’m doing better right now, I have a friend…I go to school, I like school…Me I want to change life, everything because I don’t like my country…but here beautiful…people help refugee everything. That’s the thing, and you can study, you can learn English.”* [Participant #1, Male, Iraq].

## Discussion

In this qualitative analysis of survivors of torture in Western New York, we found that self-reported mental health challenges were pervasive, and that separation from family and community contributed greatly to these challenges. The uncertainty of asylum seeker’s legal status/position in the United States and their inability to work and be productive seemed to intensify these challenges.

Refugees and asylum seekers face significant challenges during the processes of resettlement and asylum application, respectively. A 2006 report from the Robert Wood Johnson Foundation found that refugees and immigrants encountered barriers in the educational, employment and housing systems; many felt isolated and were at times subject to prejudice and discrimination [13]; findings which are echoed by the participants in this study, particulary as they relate to feelings of isolation. A 2017 study examined the effects of “media frames” about and attitudes toward refugee resettlement in the United States [14]. These authors found that anti-resettlement rhetoric was heightened by media reports, while “rebutting the threat posed by refugees” had no significant effect. In the year leading up to our study, immigration reform was fueled by anti-immigrant speechmaking as the U.S. headed into its national election, which may have contributed to participants’ feelings of isolation.

Problems for refugees also exist in timely access to general primary care, and provision of culturally aware and consistent mental health treatment [15]. While refugees enter the U.S. covered for health insurance for up to 8 months [16]; asylum seekers are not provided access to the insurance marketplace until they have been qualified for employment authorization [17]. Our participants expressed difficulties with navigating the various service systems in the United States, challenges with the wait times required to obtain work authorization, and a lack of ability to advocate for themselves. Care coordinators at the WNYCST were instrumental in assisting these survivors with access to services, and understanding the systems in the U.S.

In a study assessing 23 Torture Survivor Centers across the U.S., differences were found between refugees and asylum seekers in mental health and other social status factors [18]. Asylum seekers were more likely to have PTSD and major depressive disorder based on a higher incidence of experienced torture. Our findings likewise suggest increased difficulties with mental health among asylum seekers, based upon participants’ self-report. This indicates that asylum seekers may need different types of support and assistance to help ameliorate these challenges.

Our respondents also reported facing isolation and loss of a sense of purpose in life. They did see that the services offered through the WNYCST were helpful, particularly the assistance and connection with care coordinators and local support groups. This care coordination and outreach helped to mitigate feelings of separation and apartness from their home countries, families and communities. Qualitative evidence suggests that creating informal “communities of healing;” support groups where survivors are able to share their experiences, may help survivors to heal and to reestablish connections to others in their new communities [19]. Helping all survivors of torture to cope with separation from family and connect with others in their new communities are important components of programs that serve this population.

## Limitations

First, this study was designed to be an exploratory qualitative inquiry to describe the range of experiences encountered by survivors of torture living in Western New York. The sample included a small sample of both refugees and asylum seekers, and may not be representative of all asylum seekers and refugees. Because of the small sample and the diversity among participants, data saturation may not have been reached and other themes and factors not identified here may also be important. Future studies should consider larger samples, designed to compare findings across different subgroups among torture survivors. While the qualitative design did not allow for statistical comparisons, identified areas of need in the two groups can be examined further in on-going evaluation that will seek to quantitatively determine specific variations in unmet needs and coping strategies. Additionally, participants were all clients of the WNYCST, indicating that they have self-identified as survivors of torture and sought help. This may not be representative of all torture survivors generally. Finally, due to the limitations on the study scope, we were not able to assess the quality of the interviews across interpreters and languages; however, it is important to note that all interpreters were vetted by the WNYCST and were regularly relied upon in staff communication with clients. Nonetheless, findings will help to shape service needs and approaches going forward for organizations that serve these populations.

## Conclusion

Some refugees and asylum seekers continue to struggle with unmet needs and issues of loss and isolation. It is important that care providers recognize these signs of stress early, so that appropriate interventions can be implemented. Current anti-resettlement rhetoric may exacerbate feelings of displacement [20]. Care coordination, and trauma informed treatment with an emphasis on recreating connections with families and communities, may be one important factor in ensuring successful integration of individuals and families.

## Data Availability

The datasets used and/or analyzed during the current study are available from the corresponding author on reasonable request.
